# Editorial: Improving neurosurgical precision: leveraging technology for enhanced visualization and navigation of complex neuroanatomy

**DOI:** 10.3389/fnana.2026.1982557

**Published:** 2026-09-25

**Authors:** Georgios P. Skandalakis, Christos Koutsarnakis, Linton T. Evans, Jennifer Hong, Nathan E. Simmons, Andres M. Lozano, Aristotelis Kalyvas

**Affiliations:** 1 Department of Neurosurgery, University of Utah, Salt Lake City, UT, United States; 2 Neurosurgical Department, Red Cross, General Hospital of Athens, Athens, Greece; 3 Department of Surgery, Section of Neurosurgery, Dartmouth Health, Lebanon, NH, United States; 4 Division of Neurosurgery, Department of Surgery, University of Toronto, Toronto, ON, Canada; 5 Neurosurgical Department, Attikon University Hospital, Athens, Greece

**Keywords:** augmented reality, image guided surgery, neuronavigation, neurosurgery, surgical neuroanatomy, white matter anatomy

Neurological surgery has always been defined by the interplay between anatomical knowledge and technological innovation. From the earliest cortical maps to modern connectomic atlases, advances in our understanding of neuroanatomy have consistently catalyzed improvements in surgical safety and efficacy. This Research Topic, “*Improving neurosurgical precision: leveraging technology for enhanced visualization and navigation of complex neuroanatomy*,” was conceived to capture this dynamic relationship at a pivotal moment—one in which emerging imaging modalities, computational tools, and intraoperative technologies are converging to reshape how neurosurgeons visualize, plan, and execute procedures across all subspecialties.

The 23 articles collected here, contributed by 181 authors from institutions worldwide, examine how anatomical knowledge and technological developments can inform neurosurgical planning and execution. Anatomical investigations, including characterization of the oculomotor nerve's arterial supply, and a preclinical glioma surgery model provide complementary foundations for studying surgical risks related to specific anatomy. Beyond new anatomical findings, these studies highlight technological innovations aimed at enhancing neurosurgical precision, including virtual reality–based planning of minimally invasive aneurysm approaches, ultrasound and augmented or mixed reality guidance for glioma resection, and patient-specific localization of pallidal DBS contacts.

Clinical and methodological contributions address the translation of imaging into procedural guidance through augmented reality for glioma resection, robot-assisted stereotactic biopsy, and 3D-printed navigation templates. Work on spinal tumor visualization and tissue-specific dissection extends this scope to intraoperative tissue assessment and surgical exposure. Together, these contributions link anatomical characterization with patient-specific visualization, targeting, and operative technique, while identifying questions of accuracy, feasibility, and clinical validation. The following sections organize these complementary contributions around their principal anatomical and technological themes.

## Neuroanatomy as the foundation: laboratory investigations and white matter architecture

A central premise of this Research Topic is that technological innovation without anatomical grounding is insufficient. Several contributions reinforce this principle through cadaveric dissection, advanced tractography, and morphological analysis. These studies continue a long tradition of laboratory neuroanatomy that remains indispensable to neurosurgical training and discovery (Kshettry et al., 2014). The study of white matter connectivity has experienced a renaissance driven by diffusion-weighted imaging and fiber microdissection techniques. These complementary approaches have proven capable of revealing previously unrecognized structural substrates of brain networks—from identifying the white matter underpinnings of cortical action networks within the central sulcus, to uncovering long-range axonal connections between the precuneus and temporal pole through cingulum pathways, to characterizing the morphology and connectivity of individual cingulum bundle components relevant to surgical approaches near the posterior cingulate cortex (Skandalakis et al., 2025, 2024a, 2020). Microdissection-based mapping has similarly advanced our understanding of subcortical circuit architecture, providing granular structural evidence of the connectivity patterns that mediate the effects of deep brain stimulation (Skandalakis et al., 2024b). These examples illustrate how white matter dissection studies—whether cadaveric, computational, or hybrid—continue to generate anatomical knowledge with direct surgical relevance. Several articles within this Research Topic echo these themes by presenting novel white matter dissections, tractographic reconstructions, and morphometric analyses that expand the anatomical knowledge base upon which technology-guided surgery depends.

## Preoperative planning: advanced imaging and computational tools

Accurate preoperative visualization is the first step toward surgical precision. Multiple contributions to this Topic address innovations in preoperative planning, including functional MRI paradigms, diffusion tensor imaging protocols, and computational modeling approaches that translate complex anatomical data into actionable surgical plans. The integration of tractography into preoperative workflows has demonstrated measurable benefits in reducing postoperative neurological deficits, particularly in tumor and epilepsy surgery (Guberman et al., 2025). Technological advancements including trajectory planning through pre-operative mapping and minimally invasive surgical approaches have improved the safety and precision of operative neuro-oncology, while innovations in intraoperative tumor mapping continue to enhance surgical outcomes (Rennert et al., 2016). Several articles in this Research Topic present novel pipelines for patient-specific tract reconstruction, functional localization, and risk stratification that bring these tools closer to routine clinical implementation. The work on three-dimensional reconstruction and segmentation of patient-specific anatomy featured in this Topic further illustrates how computational tools can distill complex imaging datasets into intuitive models that enhance spatial understanding prior to surgery.

## Intraoperative technologies: augmented reality, neuronavigation, and robotics

The translation of preoperative plans into the operative field remains one of the most challenging steps in neurosurgery. Contributions to this Topic address this challenge through multiple technological platforms, including augmented reality (AR), exoscopic visualization, robotic-assisted surgery, and advanced neuronavigation systems.

AR technology, which overlays digital anatomical information onto the surgeon's real-time view of the operative field, has shown promise in enhancing intraoperative visualization across cranial and spinal procedures (Chidambaram et al., 2021; van Doormaal and van Doormaal, 2024). Several articles in this Research Topic present clinical implementations of AR and extended reality systems that demonstrate improved anatomical understanding and lesion localization during surgery. The integration of connectomic data into these platforms represents a particularly exciting frontier, enabling surgeons to visualize not only structural anatomy but also the functional networks that must be preserved.

Robotic-assisted surgery, both cranial and spinal, is another rapidly evolving domain represented in this Topic. Contributions exploring optimal trajectory selection and navigation accuracy highlight how neuroanatomical knowledge directly informs robotic planning algorithms. The case report on integrated management of a giant cervicothoracic malignant peripheral nerve sheath tumor with multimodal guidance exemplifies the clinical value of combining multiple technologies such as imaging, navigation, and surgical planning in a complex anatomical scenario. The demonstrated value of neuronavigation extends to CSF diversion procedures as well. Prospective multicenter studies illustrate how standardized, technology-augmented approaches can improve shunt outcomes in pediatric hydrocephalus (Kestle and Riva-Cambrin, 2019).

## Deep brain stimulation and functional neurosurgery

The field of deep brain stimulation (DBS) has increasingly embraced connectomic approaches, recognizing that stimulation effects propagate through white matter tracts to modulate distributed brain networks rather than isolated nuclei (Middlebrooks et al., 2020). This paradigm shift places neuroanatomy and particularly white matter architecture at the center of DBS target selection and programming. Microdissection-based mapping of midbrain stimulation-related circuits has contributed to this framework by providing the structural foundation for understanding how stimulation at subcortical targets engages cortical and subcortical networks (Skandalakis et al., 2024b). Anatomical characterization of potential neuromodulation targets such as the habenula at the convergence of functional neuroanatomy, psychiatry, and neuroimaging further underscores how precise knowledge of a target's structural connections is a prerequisite for safe and effective neurostimulation (Skandalakis et al., 2018). As DBS technology evolves, practical considerations such as electromagnetic interference (EMI) with implanted devices also warrant attention; (Rahimpour et al. 2021) have provided a comprehensive review of both unintentional and intentional sources of EMI affecting DBS systems, with implications for patient safety protocols and device design. Articles within this Topic contribute to this evolving framework by presenting novel approaches to tractography-guided targeting and outcome prediction in functional neurosurgery.

## Skull base surgery: where anatomy meets precision

Skull base neurosurgery exemplifies the indispensable role of detailed neuroanatomy in guiding technological application. The complex three-dimensional relationships among cranial nerves, vascular structures, and bony anatomy at the skull base demand precise anatomical knowledge that informs approach selection, extent of resection, and reconstruction strategies. (Raheja and Couldwell 2020) have contributed extensively to this understanding, including the management paradigm for cavernous sinus meningiomas illustrating how the pendulum has shifted from aggressive resection toward maximal safe resection with adjuvant radiotherapy, driven by improved anatomical understanding and multimodal treatment integration. Collaborative work on spheno-orbital meningiomas further demonstrates how detailed characterization of tumor behavior at the skull base interface informs surgical strategy (Rawanduzy et al., 2023). Anatomical variants can play an important role to surgical planning for safer outcomes (Skandalakis et al., 2019). Several contributions to this Research Topic present cadaveric and imaging-based investigations of skull base anatomy, complemented by clinical studies that showcase how this knowledge is translated into safer operative corridors through modern visualization and navigation technologies.

## Epilepsy surgery and neuroanatomy-guided interventions

The precision demanded in epilepsy surgery where the goal is seizure freedom without compromise to eloquent function represents another compelling application of neuroanatomy-guided technology. (Botros et al. 2026) have outlined how successful epilepsy surgery relies on accurate identification of the epileptogenic zone through comprehensive pre-surgical evaluation, including advanced imaging modalities and invasive monitoring, with extent of resection consistently identified as the critical determinant of outcome. Contributions to this Topic that address stereoelectroencephalography planning, cortical mapping, and disconnection procedures reflect the growing integration of advanced imaging and navigation technologies into the epilepsy surgery workflow.

## Surgical training and anatomical education

Several contributions address the critical role of neuroanatomy in surgical education. Hybrid training models that combine simulation with cadaveric dissection have demonstrated superior anatomical learning outcomes compared to either approach alone. The articles on training platforms and educational innovations in this Research Topic reflect a consensus that mastery of three-dimensional neuroanatomy whether acquired through the dissection laboratory, virtual reality, or both, remains essential for the development of technically proficient neurosurgeons.

## Tissue characterization and precision techniques

The contribution on precision dissection strategy for myocutaneous flaps based on tissue biophysical properties illustrates how anatomical precision extends beyond the nervous system itself. Understanding the biomechanical and structural properties of tissues encountered during neurosurgical approaches including scalp, muscle, and bone can inform reconstructive strategies that optimize both functional and aesthetic outcomes.

## Future directions

The articles in this Research Topic collectively point toward several converging trends. First, the increasing resolution of both anatomical knowledge and imaging technology is enabling truly patient-specific surgical planning. Second, the integration of multimodal data including structural connectivity, functional mapping, and intraoperative imaging into unified platforms promises to reduce the cognitive burden on surgeons while improving decision-making. Third, advances in artificial intelligence and machine learning are poised to automate and refine many of the processing steps that currently limit the clinical adoption of advanced neuroimaging techniques.

However, significant challenges remain. Standardization of tractography protocols, validation of AR registration accuracy in the setting of brain shift, and demonstration of improved patient outcomes in prospective trials are all necessary before these technologies achieve widespread adoption. The articles in this Research Topic, while advancing the field substantially, also highlight these gaps and provide a roadmap for future investigation.

## Concluding remarks

This Research Topic demonstrates that the relationship between neuroanatomy and technology in neurosurgery is not unidirectional but synergistic. Technology enables deeper anatomical discovery, which in turn drives more sophisticated technological applications. The 23 contributions assembled here spanning cadaveric dissection, advanced neuroimaging, intraoperative visualization, robotics, and surgical education collectively affirm that neuroanatomy remains the indispensable foundation upon which neurosurgical innovation is built. We are grateful to all contributing authors for their rigorous work and to the reviewers and editorial staff who ensured the quality of this Research Topic.
